# Time to death and its associated factors among infants in sub-Saharan Africa using the recent demographic and health surveys: shared frailty survival analysis

**DOI:** 10.1186/s12887-021-02895-7

**Published:** 2021-10-04

**Authors:** Sofonyas Abebaw Tiruneh, Ejigu Gebeye Zeleke, Yaregal Animut

**Affiliations:** 1 Department of Public Health, College of Health Sciences, Debre Tabor University, Debre Tabor, Ethiopia; 2 Department of Epidemiology and Biostatistics, Institute of Public Health, College of Medicine and Health Sciences, University of Gondar, Gondar, Ethiopia

**Keywords:** Infant mortality rate, Shared frailty, SSA

## Abstract

**Background:**

Globally, approximately 4.1 million infants died, accounting for 75% of all under-five deaths. In sub-Saharan Africa (SSA), infant mortality was 52.7/1000 live births in 2018 This study aimed to assess the pooled estimate of infant mortality rate (IMR), time to death, and its associated factors in SSA using the recent demographic and health survey dataset between 2010 and 2018.

**Methods:**

Data were retrieved from the standard demographic and health survey datasets among 33 SSA countries. A total of 93,765 samples were included. The data were cleaned using Microsoft Excel and STATA software. Data analysis was done using R and STATA software. Parametric shared frailty survival analysis was employed. Statistical significance was declared as a two-side *P*-value < 0.05.

**Results:**

The pooled estimate of IMR in SSA was 51 per 1000 live births (95% Confidence Interval (CI): 46.65–55.21). The pooled estimate of the IMR was 53 in Central, 44 in Eastern, 44 in Southern, and 57 in Western Africa per 1000 live births. The cumulative survival probability at the end of 1 year was 56%. Multiple births (Adjusted Hazard ratio (AHR) = 2.68, 95% CI: 2.54–2.82), low birth weight infants (AHR = 1.28, 95% CI: 1.22–1.34), teenage pregnancy (AHR = 1.19, 95 CI: 1.10–1.29), preceding birth interval <  18 months (AHR = 3.27, 95% CI: 3.10–3.45), birth order ≥ four (AHR = 1.14, 95% CI:1.10–1.19), home delivery (AHR = 1.08, 95% CI: 1.04–1.13), and unimproved water source (AHR = 1.07, 95% CI: 1.01–1.13), female sex (AHR = 0.86, 95% CI: 0.83–0.89), immediately breastfeed (AHR = 0.24, 95% CI: 0.23–0.25), and educated mother (AHR = 0.88, 95% CI: 0.82–0. 95) and educated father (AHR = 0.90, 95% CI: 0.85–0.96) were statistically significant factors for infant mortality.

**Conclusion:**

Significant number of infants died in SSA. The most common cause of infant death is a preventable bio-demographic factor. To reduce infant mortality in the region, policymakers and other stakeholders should pay attention to preventable bio-demographic risk factors, enhance women education and improved water sources.

**Supplementary Information:**

The online version contains supplementary material available at 10.1186/s12887-021-02895-7.

## Introduction

Infant Mortality Rate (IMR) is the death of an infant before celebrating the first birthday per thousand live births in a given population [1]. Infant mortality is a highly sensitive indicator of the present health condition as well as a prediction of future health conditions [1–4]..

Globally in 2017, approximately 4.1 million deaths occurred within the first year of life, accounting for 75% of all under-five deaths [5]. In SSA, infant mortality was 52.7 per 1000 live births in 2018, which is unacceptably high as compared to other regions [6]. According to the 2019 United Nations Inter-Agency Group for Child Mortality Estimation (UN-IAME) report, infant mortality in SSA ranges from 28.2 (Rwanda, the lowest) to 86.5 (the Central Africa Republic, the highest) per 1000 live births (8). In SSA, one in 36 children dies during the neonatal period, as compared with 1 in 333 in developed nations [7]. Globally, the IMR has declined from 53.2 per 1000 live births in 2000 to 28.9/1000 live births in 2018 [8]. Even though infant mortality significantly declined worldwide, the decline in SSA was unsatisfactory which, is 92/1000 live births in 2000 to 53/1000 live births in 2018 [6].

The United Nations (UN) Sustainable Development Goal (SDG) 3 target 3.2 calls reducing under-five mortality to at least 25/1000 live births [9]. In line with this target, infant mortality accounts for a large share (75%) of under-five deaths [10]. Most SSA countries are not on track to neonatal and under-5 mortality targets regarding the SDG-3 by 2030 [11]. Only five countries (Kenya, Rwanda, Senegal, Tanzania, and Uganda) are on track to decline the under-5 mortality SDG target by 2030, but only Rwanda and Tanzania would meet the neonatal mortality targets [11].

Even though most SSA countries go on off-track to the SDG targets regarding infant death, there is a paucity of information on pooled estimates and factors affecting infant death in SSA. So far, there has been no pooled estimate regarding IMR in SSA controlling Country-level frailty using Demographic and Health Survey (DHS) datasets. Conducting and documenting the pooled estimate of IMR, time to death, and its determinants in SSA would help policymakers and health planners for each country to achieve the SDG agenda. Therefore, this study aimed to assess the pooled estimate of IMR, time to death and its associated factors among infant’s mortality in SSA.

## Methods and materials

### Data sources

The data for this study extracted from the recent standard DHS (2010–2018) datasets of 33 SSA countries. Standard Demographic and Health Surveys (DHSs) are nationally representative and population-based surveys collected through uniform questionnaires and manuals that are comparable across countries. The data were collected using multi-stage stratified, cluster sampling techniques for each country. The details of the recorded data are available at https://dhsprogram.com/.

### Populations and samples

The source population consisted of all live births preceding 5 years of the survey period across 33 SSA countries. The study populations were all live births preceding 5 years of the survey period in the selected Enumeration Areas (EAs) for each country. Data extracted from the birth record (BR) file from the standard DHS dataset of SSA countries with at least one recent survey from 2010 to 2018. A total of 93,765 infants were included from 12 East African, 7 Central African, 13 West African, and 3 South African countries. The details of the samples included for each country is available in the supplementary file (Supplementary Table A).

### Eligibility identification

All live births followed for one-year full cohort preceding 5 years of the survey in the selected EAs was included. However, countries (Central Africa Republic, Eswatini, Sao Tome Principe, Madagascar, and Sudan) did not have a DHS survey report after the 2010/2011 survey year was excluded due to the recent updates. Because the datasets are not publicly available, three SSA Countries (Botswana, Mauritania, and Eritrea) were excluded from this study.

### Study variables

#### Outcome variable

The outcome variable of this study was time to infant death in months. The survival time of an infant beyond 12 months was declared censored. Infant death between birth and 12 months was declared as an event. The outcome variable coded 0 as censored and 1 as event (0 = alive and 1 = death).

#### Independent variables

Socioeconomic and demographic variables include mothers and husband education (no education, primary, secondary and above education), household wealth index (poor, middle, rich), residence (urban, rural), latrine source (had latrine, no latrine), water source (improved, not improved), and County income(low income, lower middle income, higher middle income). Maternal reproductive and obstetric variables include preceding birth interval (≥ 24 months, 18–23 months, < 18 months), teenage pregnancy (≥ 20 years, < 19 years), place of delivery (health facility, home), antenatal care visit (no, at least one visit), and birth order (≤ three, ≥ four). Moreover, infant characteristic variables include the child sex (male, female), weight of the child at birth (average, smaller than average, larger than average), and plurality of the child (single, multiple).

### Data management and analysis

The data were cleaned, coded, and extracted using Microsoft Excel and STATA version 16/MP software. Sample weighting was performed for each country before further analysis.

#### The pooled estimate of IMR

Infants born in different cohorts do not contribute equally to the denominator of the IMR calculation. As a result, all live birth infants do not contribute equally to the infant mortality calculation in the DHS dataset. The pooled estimate of IMR in SSA was estimated using the *DHS.rates R package* in R software. The *DHS.rates R package* can estimate the point estimate of IMR with their standard error for each country [12]. After calculating the IMR with their standard error in each country (Sup. Table 2), the pooled estimate of IMR in SSA and sub-regions was estimated using a meta-analysis “*metan”* STATA command.

#### Modeling of parametric shared frailty survival analysis

The Frailty model has an unobserved multiplicative effect on the hazard rate for all individuals in the same group. In the shared frailty model infants in the same Country share the same nuisance (frailty) factor. Parameter *θ* provides information on the variability (dependency) of the population in the same Country. Infants in Country i with u_i_ > 1 and u_i_ < 1 have a more frail than higher risk and lower risk respectively. Based on the different frailty term one frailty term was employed using Country taken as random effect dependency. For a single frailty term, the model specification is given by [13].

$${\mathrm{h}}_{\mathrm{i}\mathrm{j}\left(\mathrm{t}\right)}={\mathrm{h}}_0\left(\mathrm{t}\right){\mathrm{u}}_{\mathrm{i}}\ \exp .\left({\mathrm{x}}_{\mathrm{j}}^{\mathrm{i}}\upbeta \right)$$

where u_i_ = exp.(wi) is the frailty for the i^th^ Country. u_i_’s, i = 1,. .., s, are the actual values of a sample from density *f*_*U*_.

The parametric frailty model fitted using the Gompertz baseline hazard distributional assumption and the gamma frailty distribution model fitted in Country taken as random effects frailty for the independent variables.

#### Level of dependence in the shared frailty model

The correlation between any two event times from the same country was measured using Kendall’s tau (τ). Kendall’s tau (τ) measured the dependency of two events in the same country, dividing the frailty (*θ*) by two-plus frailty(*θ*). The higher the frailty(*θ*) the higher dependency and the higher Kendall’s tau (τ) [14].

$$\mathrm{Kendall}'\mathrm{s}\ \mathrm{tau}\ \left(\uptau \right)=\frac{\theta }{\theta +2},\mathrm{where}\ \uptau\ \upvarepsilon\ \left(0,1\right)$$

#### The best fit model selection

The best fit model was selected using Akakian Information Criteria (AIC) and the Log-likelihood ratio test. The lowest Akakian Information Criteria and the highest Log-likelihood ratio value indicates the best fit model. The Cox-Snell residual plot also employed for the model diagnosis. If the model fits the data well, the Cox-Snell residuals should have a standard exponential distribution with λ = 1. One way to verify the fit is to calculate an empirical estimate of the cumulative hazard function based on the Kaplan–Meier survival estimates taking the Cox–Snell residuals as the time variable. If the model fits the data, the plot should be a straight line with a slope of 1.

### Ethical consideration

This study was performed under the ethical standards of the Helsinki declaration and its subsequent amendments. The ethical clearance approved by the University of Gondar Institutional Review Board (IRB) (ref:  091/26/1006/12). As well, a waiver of written informed consent secured from the International Review Board of Demographic and Health Surveys (DHS) program data archivists to download the dataset for this study. The dataset was not shared or passed on to other bodies.

## Results

### Background characteristics of the study respondents

The mean age of the mother was 28 (± 7 SD) years and, most (74%) of the mothers were in the age group of 20 to 34 years. The majority (92%) of the mothers were married. Most (71%) of the respondents were rural inhabitants. Eighty per cent of the households headed by males and more than three-fourth (78%) of the households used an open water source. Among SSA countries, 60% of them were low-income countries (Table 1).

**Table 1:** Background characteristics of the study respondents in SSA, using the recent DHS 2010 to 2018, 2020

Variables	Categories	Frequency (n)		Unweighted percentage
		Unweighted	Weighted	
Maternal age	15–19	10,648	10,468	11.36
	20–34	69,081	68,883	73.67
	35–49	14,036	13,625	14.97
Age of the mother at birth	≥ 20 years	78,236	15,249	83.44
	≤ 19 years	15,529	77, 228	16.56
Marital status	Not currently married	7188	6784	7.67
	Married	86,577	86,192	92.33
Mother educational status	No education	39,563	38,608	42.19
	Primary	30,602	30,254	32.64
	Secondary and above	23,600	24,115	25.17
Mother occupational status	Not-working	29,690	29,037	33.96
	Working	57,749	57,520	66.04
Husband educational status	No education	31,206	30,759	39.25
	Primary	21,642	21,785	27.22
	Secondary and above	26,663	26,735	33.53
Husband occupational status	Not-working	2774	2716	3.48
	Working	77,020	77,065	96.52
Residence	Urban	27,524	28,441	29.35
	Rural	66,241	64,536	70.65
Head of household	Male	75,037	75,080	80.03
	Female	18,728	17,896	19.97
Wealth status	Poor	45,483	42, 130	48.51
	Middle	18,514	18,812	19.75
	Rich	29,768	32,056	31.75
Latrine facility	Had latrine	61,105	62,427	65.17
	No latrine	32,656	30,543	34.83
Water source	Improved	20,799	21,727	22.18
	Unimproved	72,966	71,250	77.82
Country income	Low income	55,585	56,266	59.28
	Lower middle income	34,548	33,348	36.85
	Higher middle income	3632	3362	3.87
Sub-Saharan region	East Africa	30,065	30,176	32.06
	West Africa	40,727	40,494	43.44
	Central Africa	20,001	19,423	21.33
	Southern Africa	2972	2885	3.17
**Total**		**93,765**	**92,977**	**100**

### Infant characteristics

Of the total infants, 48,471 (52%) were males. Ninety-five per cent of infant births were singleton. Only 59,480 (82%) of infants were born with a preceding birth interval greater than 2 years. Twenty per cent of infants had been low birth weight. Only 40% of infants were immediately breastfed at birth. Moreover, 57,416 (63%) infants were delivered at the health facility. Only 4785 (5%) of them give birth by cesarean section (Table 2).

**Table 2:** Infant characteristics of SSA countries using the recent DHS 2010 to 2018, 2020

Variables	Categories	Frequency (n)		Unweighted percentage
		Unweighted	Weighted	
Infant sex	Male	48,471	48,076	51.69
	Female	45,294	44,901	48.31
Plurality	Single	88,605	87,882	94.50
	Multiple	5160	5095	5.50
Preceding birth interval	≥ 24 months	59,480	58,989	81.58
	18–23 months	8586	8356	11.78
	< 18 months	4848	4727	6.65
Birth order	≤ three	52,202	52,380	55.67
	≥ four	41,563	40,597	44.33
Birth size at birth	Smaller than average	16,921	16,797	19.54
	Average	39,310	39,067	45.40
	Larger than average	30,356	30,148	35.06
Duration of breastfeeding	Never breastfed	26,947	26,836	28.74
	Still breastfeeding	66,818	66,141	71.26
Breastfeeding at birth	Not-immediately	56,278	55,455	60.02
	Immediately	37,487	37,522	39.98
ANC visits	No ANC	23,791	23,369	25.37
	At least one ANC	69,974	69,608	74.63
Place of delivery	Health facilities	57,416	57,714	63.13
	Home delivery	33,533	32,459	36.87
Mode of delivery	SVD	88,980	87,970	94.90
	Cesarean section	4785	5006	5.10
**Total**		**93,765**	**92,977**	**100**

### The pooled estimate of IMR in SSA

**Fig. 1:**
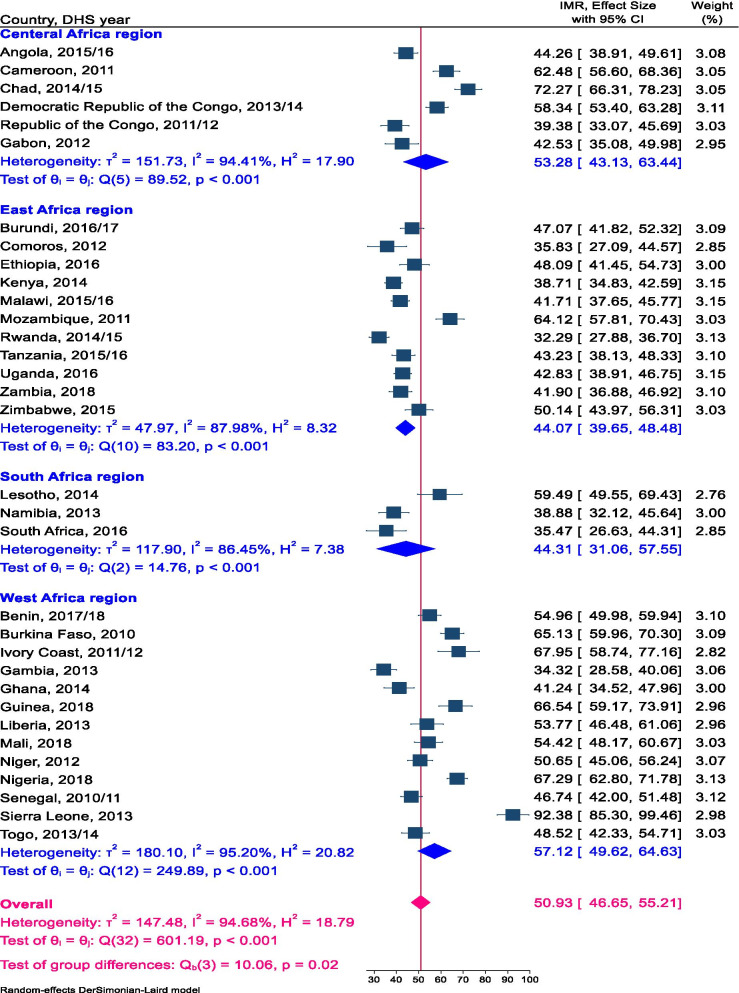
Pooled estimate of IMR in Sub-Sharan Africa using the recent DHS 2010 to 2018, 2020

Overall, 361,826 weighted number of live births contributed to the denominator to the calculation of the pooled estimate of IMR across 33 SSA countries. The pooled estimate of IMR across 33 SSA countries was 51 per 1000 live births (95% CI: 46.65–55.21). The pooled estimate of IMR was 53 per 1000 live births (95% CI: 43.13–63.44) across six Countries of Central Africa region, 44 per 1000 live births (95% CI: 39.65–48.48) in 11 Countries of the East Africa region, 44 per 1000 live births (95% CI: 31.06–57.53) among three South African Countries, and 57 per 1000 live births (95% CI: 49.62–64.63) across 13 West African Countries (Fig. 1). The pooled estimate of IMR in urban inhabitants was 45 per 1000 live births (95% CI: 40.63–49.64) (Sup. Fig. A) across 31 sub-Saharan Africa countries; whereas in rural inhabitants were 53 per 1000 live births (95% CI: 47.85–57.86) (Sup. Fig. B). The pooled estimate of IMR in low-income countries was 52 per 1000 live births (95% CI: 45.92–58.41), 51 per 1000 live births in lower-middle-income countries (95% CI: 43.07–57.39), and 46 per 1000 live birth across high middle-income countries (95% CI: 32.07–59.76) (Sup. Fig. C).

### Descriptive survival analysis

#### Infant’s survival in SSA

From a total of 93,765 infants, 20,865 infants were died before celebrating their first birthday. The cumulative survival probability of infants at the end of 1 year was 56% (95% CI: 0.55–0.57). The cumulative survival probability of surviving at the end of 6 months was 81%. The cumulative survival probability at the end of 1 year was 55% among males and 58% among females (Table 3).

**Table 3:** Life time table cumulative survival probability of infants in SSA using the recent DHS 2010 to 2018, 2020

Time after birth	Cumulative survival probability % (95% CI)		
	Male	Female	Total
Up to first month	85.76 (85.45–86.08)	89 (88.70–89.29)	87.33 (87.11–87.54)
Third month	82.58 (82.23–82.92)	86.22 (85.89–86.54)	84.34 (84.10–84.57)
Six months	78.68 (78.29–79.07)	82.48 (82.10–82.85)	80.52 (80.24–80.79)
Nine months	72.23 (71.73–72.72)	76.01 (75.51–76.51)	74.05 (73.70–74.41)
One year	55.11 (53.85–56.35)	57.81 (56.44–59.16)	56.41 (55.49–57.33)

#### Kaplan Meier survival analysis

**Fig. 2:**
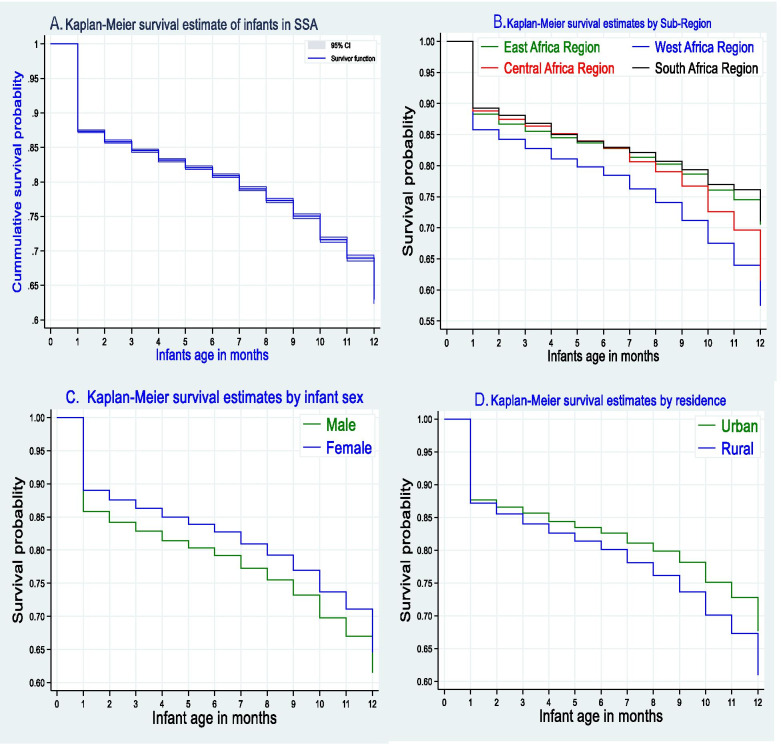
Overall Kaplan Meier survival estimate of infants (Fig. **A**), survival estimates in SSA Africa sub-regions (Fig. **B**), survival estimates by sex of infants (Fig. **C**), and survival estimates of infants by residence (Fig. **D**)

The survival probability of the infants was estimated using the non-parametric Kaplan Meier survival estimate. The probability of death in the first month of life was high. After the first month of life, the probability of survival of infants was decreased proportionally (Fig. 2A). The probability of death among infants in the West Africa region was higher than in the rest of the regions and after the age of 7 months the probability of death was high among infants live in the Central Africa region. Whereas, the survival probability of infants in East and Southern African region were proportional through one-year life (Fig. 2B). Male infants have a high probability of death as compared to female infants. Infants living in rural areas had a high probability of death than their counterparts (Fig. 2).

### Model comparison

Based on the information criteria Gompertz baseline distribution was the best fit model with gamma frailty distribution. The Log-logistic and Lognormal baseline hazard distribution model and the inverse gaussian frailty distribution models did not converge (Table 4).

**Table 4:** Model comparison with different distributional assumptions

Model	Baseline hazard distribution	Frailty distribution	Frailty variance (θ, p-value)	AIC	BIC	LRR
Cox-model	NA	Gamma	0.05, < 0.001	239,411	239,609	− 119,683
Shard frailty	Exponential	Gamma	0.06, < 0.001	76,330.47	76,247.13	−38,141.24
Shard frailty	Weibull	Gamma	0.06, < 0.001	76,168.05	76,393.73	−38,059.24
Shard frailty	Gompertz	Gamma	0.06, < 0.001	76,017.40	76,243.08	−37,983.70
Shard frailty	Log-logistic	Gamma		The model did not converge		
Shard frailty	Lognormal	Gamma				

NB: Model with inverse Gaussian frailty distribution did not converge

NB: AIC = Akakian Information Criteria, BIC = Bayesian Information Criteria, LLR = Loglikelihood Ratio, NA = Not Applicable

### Factors affecting infant mortality in SSA

To identify the potential significant factors for infant mortality country-level parametric shared frailty survival model was fitted. The value of the shape parameter in the Gompertz baseline hazard distribution model was (ρ = − 0.06. 95% CI: − 0.07 – -0.05). The negative shape parameter indicates the hazard of death among infants were decreased exponentially as the age of infants increases. The dependency (heterogeneity) of infant death in the same Country estimated by the model was statistically significant with a value theta (θ) = 0.06 (θ = 0.06, 95% CI: 0.04–0. 11), and the dependence within-country was τ = 3% (95% CI: 0.02–0.05) which the lowest dependency was 2% and the highest 5% across the country.

After controlling country-level frailty, the results from Gompertz parametric baseline hazard distribution revealed that infant, maternal reproductive and obstetrics, and socioeconomic characteristics were statistically significant predictors for infant survival (Table 5).

**Table 5:** Results of multivariable parametric Gompertz distribution country-level shared frailty survival regression model among infants in SSA countries, 2020

Variables	Categories	Infant status		CHR (95%CI)	AHR (95%CI)
		Alive	Dead		
Infant sex	Male	36,795	11,676	1	1
	Female	36,105	9189	0.82 (0.80–0.84)	0.86 (0.83–0.89) ***
Plurality	Single	70,550	18,055	1	1
	Multiple	2350	2810	3.47 (3.34–3.62)	2.68 (2.54–2.82) ***
Birth size at birth	Average	32,279	7031	1	1
	Smaller than average	12,449	4472	1.61 (1.55–1.67)	1.28 (1.22–1.34) ***
	Larger than average	25,015	5341	0.95 (0.91–0.98)	0.95 (0.91–0.99) *
Immediately breastfeed	No	38,106	18,172	1	1
	Yes	34,794	2693	0.18 (0.17–0.19)	0.24 (0.23–0.25) ***
Teenage pregnancy	≥ 20 years	61,623	16,613	1	1
	< 19 years	11,277	4252	1.31 (1.27–1.36)	1.19 (1.10–1.29) ***
Birth interval	≥ 24 months	49,008	10,472	1	1
	18–23 months	5732	2854	2.02 (1.94–2.12)	1.93 (1.83–2.02) ***
	< 18 months	2363	2485	3.57 (3.41–3.73)	3.27 (3.10–3.45) ***
Birth order	≤ Three	41,166	11,036	1	1
	≥ Four	31,734	9829	1.10 (1.07–1.13)	1.14 (1.10–1.19) ***
Place of delivery	Health facility	47,266	10,150	1	1
	Home	25,624	7909	1.31 (1.27–1.35)	1.08 (1.04–1.13) **
Mode of delivery	SVD	69,097	19,883	1	1
	Cesarean section	3803	982	1.02 (0.96–1.09)	1.02 (0.94–1.12)
Mother education	No education	29,303	10,260	1	1
	Primary	24,126	6476	0.91 (0.88–0.95)	0.99 (0.94–1.04)
	Secondary & above	19,471	4129	0.69 (0.66–0.72)	0.88 (0.82–0.95) ***
Father education	No education	22,966	8240		1
	Primary	16,988	4654	0.91 (0.87–0.95)	0.95 (0.90–0.99) **
	Secondary & above	21,475	5188	0.75 (0.72–0.78)	0.90 (0.85–0.96) ***
Residence	Urban	21,998	5526	1	1
	Rural	50,902	15,339	1.15 (1.11–1.18)	1.04 (0.98–1. 09)
Wealth index	Poor	34,814	10,669	1	1
	Middle	14,313	4201	0.94 (0.91–0.97)	1.03 (0.96–1.07)
	Rich	23,773	5995	0.82 (0.80–0.85)	1.04 (0.98–1.10)
Latrine facility	Had latrine	47,865	13,240	1	1
	No latrine	25,033	7623	1.10 (1.07–1.14)	1.04 (0.99–1.09)
Water source	Improved	16,954	3845	1	1
	Not improved	55,946	17,020	1.16 (1.11–1.20)	1.07 (1.01–1.13) *
Country income	Low income	42,743	12,842	1	1
	Lower middle income	27,115	7433	0.87 (0.71–1.01)	0.96 (0.80–1.17)
	Higher middle income	3042	590	0.70 (0.48–1.02)	1.12 (0.79–1.59)
**Gompertz distribution shape parameter gamma (γ)**					**−0.06 (−0.07** – **−0.05)**
**Frailty theta (θ)**					0.06 (0.04–0.11) ***
**Kendall’s tau (τ)**					**0.03 (0.02–0.05)**

NB: * = significant at 0.05 level, ** = significant at 0.01 level, *** = significant at 0.001 level, CHR = Crude Hazard Ratio, and AHR = Adjusted Hazard Ratio

Infant sex, plurality, birth weight, and immediate breastfeeding status after birth had a statistically significant association with infant survival. The estimated hazard of death among female infants was lower by 14% as compared to male infants (AHR = 0.86, 95% CI: 0.83–0.90). The risk of death among multiple-birth infants was 2.68 times higher than singleton birth infants (AHR = 2.68, 95% CI: 2.54–2.82). The hazard of infant death among smaller than average birth weight infants at birth was higher by 28% than the average birth size at birth (AHR = 1.28, 95% CI: 1.22–1.34). Infants with larger than average birth weights were 5% less likely to die than the reference category (AHR = 0.95, 95% CI: 0.91–0.99). Moreover, the estimated hazard of death among infants who initiated immediately breastfeed after birth was lower by 66% as compared to their counterparts (AHR = 0.24, 95% CI: 0.23–0.25).

Teenage pregnancy was a risk for infant survival. Infants born from whose mothers age between 15 and 19 years were 19% more likely to die as compared to infants born from mothers older than 20 years (AHR = 1.19, 95% CI:1.10–1.29). The estimated hazard of death among infants born less than 18 months preceding birth interval was 3.27 times higher than infants born greater than 2 years preceding birth interval (AHR = 3.27, 95% CI: 3.10–3.45). As well, infants born between 18 and 23 months preceding birth interval were 93% higher risk of death as compared to the reference category (AHR = 1.93, 95% CI: 1.83–2.02). Higher birth order greater than four were higher risk of death by 14% than their counterparts (AHR = 1.14, 95% CI: 1.10–1.19). Moreover, the hazard of death among infants born at home was higher by 8% as compared to infants born at a health facility (AHR = 1.08, 95% CI:1.04–1.13).

The estimated hazard of death among infants born from an educated mother (secondary and above) was reduced by 12% as compared to infants born from non-educated mothers (AHR = 0.88, 95% CI: 0.82–0.95). Besides, infants born from an educated father who had primary education and secondary and above education level were 5 and 10% lower than infants born from non-educated fathers respectively. Furthermore, infants born from households that use unimproved water sources were 7% more likely to die than households that used unimproved water sources (Table 5).

## Discussion

Infant mortality is a major public health problem with an important contributor to under-five mortality by three-fourth in SSA [5]. Reducing infant mortality is a crucial objective to achieve the UN Sustainable Development Goals. This study aimed to determine the pooled estimates of IMR and determinants of infant mortality in SSA.

This study showed that the pooled estimate of infant mortality across 33 SSA countries was 51 per 1000 live births. Sub-regional variations also observed across the four sub-regions of SSA.

The pooled estimate of IMR in the Central Africa region was 53 per 1000 live births higher than the pooled estimate of SSA. Among the six Central Africa countries, Chad (72.27/1000 live birth) had a statistically significant highest estimate of IMR in the region. The pooled estimate of IMR in the East Africa region was 44.07 per 1000 live births lower than the pooled estimate SSA. Mozambique (64.12/1000 live birth) had the highest and Rwanda (32.29/1000 live birth) had the lowest estimate of IMR as compared to other East Africa region countries. The pooled estimate of IMR in the Southern Africa region was 44 per 1000 live births which are lower than SSA estimate but not statistically significant. Lesotho had the highest point prevalence of IMR in the South Africa region. Furthermore, the pooled estimate of IMR in the West African region (57 per 1000 live births) was higher than the rest regions of SSA. Sierra Leone had a significantly higher estimate of IMR; Whereas, Gambia and Ghana had a significantly low estimate of IMR than the West Africa region pooled estimate.

The pooled estimate of IMR in SSA was alike to the recent Inter-agency Group for Child Mortality estimation in SSA in 2018 (52.7/1000 live births) [6] whereas, higher than in European regions (8/1000 live births) [10]. Collectively, higher infant death was observed in West and Central Africa as compared to East and Southern Africa region. The possible variation across sub-regions and pooled estimate of SSA countries might be surveyed year difference. Another possible source of difference across Countries, sub-regions and the pooled estimate of SSA might be universal healthcare coverage, socioeconomic context, adoption and implementation of policies and programmes to reduce IMR.

This study revealed that infant death was reduced by 14% among female infants as compared to male infants which is supported by different kinds of literature [15–19]. Another study, the infant’s survival differences among the male and female infants were not observed [20]. The possible justification might be genetic and biological variation with the male sex being biologically weaker and more susceptible to diseases and premature death than female infants which have a biological advantage on survival during the first month of life [21]. As well, male fetus exhibits intrauterine growth retardation, premature birth, and pregnancy-induced hypertension more on female fetuses might lead to early infantile death [22].

This study evidenced that the risk of death among multiple-birth infants was higher by 2.68 times than singleton births. This finding was similar to previous studies conducted in Ethiopia [15], a birth cohort study at Guinea Bissau [18], and a study from the US National Center for Health Statistics [23]. The possible explanation for this evidence might be multi-fetal pregnancy and births lead to adverse fetal outcomes during pregnancy and childbirth [24]. Another justification might be multiple births were more than twice as likely to die from external causes such as suffocation and strangulation in bed as compared to singletons [25]; As well as parents with multiple births experience more anxiety, stress, and depression in the first year of life after birth than parents of singletons and had less attention to their child [26, 27]. Besides, multiple births increase individual family size which leads to prenatal attention per child diminishes [28].

In the present study, a higher risk of death was found among smaller than average birth weight infants which had a higher risk of death by 28% than average birth weight infants. This finding is in line with existing literatures [15, 19, 29]. Possibly small birth weight infants are more venerable to neonatal sepsis, hypoglycemia and hypothermia at birth than average birth weight infants, and more likely preterm births; which lead to more risk of death [30, 31].

Moreover, infants who had immediately breastfeeding status at birth had a lower risk of death by 66% in the infantile period which was similar to the previous studies [17, 32–34]. This finding is also in line with the previous systematic review and meta-analysis study; which shows infants who initiated breastfeed after 24 h after birth had a two-fold greater risk of mortality [35]. The explanation might be first milk colostrum is rich in immunoglobins (antibodies) that stimulate the immune system and prevent infections of the gastrointestinal tract used for infant survival [15, 36].

Teenage pregnancy had a higher risk of infant death by 19% as compared to their counterparts. Similar studies witnessed that teenage pregnancy is a risk factor for infant survival [18, 29]. Another study evidenced a strong association between young maternal age during pregnancy and high infant mortality with a high prevalence of giving low birth weight [37]. The possible explanation for this result suggests that physical and physiological immaturity and the greater likelihood of inadequate weight gain during pregnancy among teenage mothers to give birth [38]. A qualitative study in South Africa evidenced that, teenage mothers had a limited role in the infant feeding decision-making process [39]. The younger the mother, the more likely that she will be immature at birth and that her child will die [40].

This study showed that a short birth interval was a significant predictor of infant mortality. The hazard of death among infants born less than 18 months preceding birth interval was higher by 3.27 times than infants born with a birth interval of more than 2 years. As well the risk of death was also higher by 93% among infants born with a birth interval between 18 and 23 months as compared to the reference category. Different studies supported the findings of this study. Evidence from 52 Demographic and Health Surveys indicates that short birth interval had a risk of infant survival [41]. Another retrospective survey data from the Demographic and Health Surveys from 17 developing countries indicates that the risk of dying among infants decreases with increasing preceding birth interval lengths up to 36 months [20]. A systematic review and meta-analysis from Ethiopia evidenced that the risk of infant death was doubled among infants born shorter than 2 years preceding birth interval [42]. The reason for this association might be a shorter birth interval increase the risk of premature birth, low birth weight, and poor pregnancy outcome. As well, the adverse consequences of a short birth interval in infant mortality attributed to biological effects related to the maternal depletion syndrome; such as if women become pregnant again before folate restoration is complete due to short birth interval, their offspring may be at a higher risk of folate insufficiency leading to increased risks of intrauterine growth retardation, and preterm birth [43]. Since the World Health Organization recommended birth spacing to wait 2 years after a live birth before attempting a next pregnancy [33], this finding calls birth spacing in Sub-Sharan Africa as per the recommendation. Moreover, higher birth order had a risk on infant survival which was similar to different studies [18, 29]. Collectively, the possible justification might be a short birth interval and higher birth order related to the four too (too close, too early, too late, and too many pregnancies) maternal and child health problems.

Place of delivery was a significant predictor for infant survival. Infants born at the home had a high risk of death by 8% as compared to health facility birth similar to previous studies [44]. Contrary to this finding previous study reported that delivery at the health facility increase the odds of infant mortality [45] and some studies showed that giving birth at home and health facility had no survival differences among infants [15, 18]. The possible explanation might be health facility delivery could prevent pregnancy-related infant death. As well infant born at the health facility will be got appropriate delivery care, vaccination, and health care provider recommendations.

Furthermore, born from educated mothers (secondary and above) benefited by a 12% increased survival than infants born from non-educated mothers; which is similar to previous existing literatures [20, 46–48]. Possibly, mothers with a higher level of education had a better economic status, good knowledge of childcare practices, and their child’s health status. Educated mothers might have autonomy in health care and feeding practice decisions for their babies [49]. Additionally, educated fathers had a positive association with infant survival, which shares a possible explanation with mother education. Unimproved sources of water had a risk of infant death by 7% in line with previous studies from an ecological study using data from 192 countries for the period 1990–2011 [50]. Another study in SSA [51], Andhra Pradesh, India [52], and Nigeria [53] supports unimproved water source has increased the hazard of infant mortality. The finding supports the fact that children accessing an improved source of water decrease infant and childhood mortality [54].

This study follows some limitation and strengths: Since the study was conducted based on a nationally representative multi-country large dataset that could enhance the generalizability of the estimates in infant mortality in SSA. Controlling country-level dependency using a shared frailty model could give an unbiased effect size. Another strength of this study was estimating the pooled estimate of IMR in SSA and sub-regions will give invaluable information for region-specific interventions. However, the data were collected cross-sectionally at a different point in time by self-reported interview, which would be prone to recall and social desirability bias. Substantial statistically significant heterogeneity was observed in pooled estimate IMR that would affect the interpretation of the pooled estimate. The drawback of the secondary nature of data was inevitable.

## Conclusions and recommendations

Even though the IMR in SSA becomes decreasing, still a significant number of infants were dying. West and Central Africa regions had the highest infant death. The most cause of infant death is preventable bio-demographic factors such as immediate breastfeeding status, teenage pregnancy, preceding birth interval, and birth order. As well, infant sex, multiple pregnancies, place of delivery, birth weight, mother and father education, and water source were statistically significant factors for infant mortality in SSA.

To tackle infant mortality in SSA, policymakers and other stakeholders of the country should give prior attention to modifiable bio-demographic factors such as preceding birth interval, immediate breastfeeding status, and teenage pregnancy.

## Supplementary Information

**Additional file 1: Table A.** Sample size for infant survival in SSA for each country. **Table B.** Infant mortality rate with their standard error for each SSA country. **Figure A.** Forest plot of the pooled estimate of IMR in urban inhabitants across SSA countries using the recent DHSs between 2010 to 2018, 2020. **Figure B.** Forest plot of the pooled estimate of IMR in rural inhabitants across SSA countries using the recent DHS between 2010 to 2018, 2020. **Figure C.** Forest plot of the pooled estimate of IMR by country income across SSA countries using the recent DHS between 2010 to 2018, 2020.

## Data Availability

The data were publicly available datasets in the DHS program.

## Abbreviations

CHR: Crude Hazard Ratio
AHR: Adjusted Hazard Ratio
CI: Confidence Interval
DHS: Demographic and Health Survey
SSA: sub-Sharan Africa
IMR: Infant Mortality Rate
SDG: Sustainable Development Goals

## References

[1] Murphy SL, Xu JQ, Kochanek KD AE. Mortality in the United States, 2017 Key findings Data from the National Vital Statistics System. NCHS Data Brief. 2018;:1–8. https://www.cdc.gov/nchs/products/databriefs/db328.htm. 30500322

[2] Reidpath DD, Allotey P (2003). Infant mortality rate as an indicator of population health. J Epidemiol Community Health.

[3] Dube L, Taha M, Asefa H. Determinants of infant mortality in community of Gilgel gibe field research center, Southwest Ethiopia: a matched case control study. BMC Public Health. 2013;13. 10.1186/1471-2458-13-401 PMC3644261 23621915

[4] Leavitt MO. Healthy People 2000 Final Review 2007.

[5] World Health Organization (WHO). Global Health Observatory (GHO) data Infant mortality Situation and trends. 2017. https://www.who.int/gho/child_health/mortality/neonatal_infant_text/en/.

[6] World Bank (2020). Infant mortality rate for developing countries in sub-Saharan Africa [SPDYNIMRTINSSA], retrieved from FRED.

[7] World news. Combatting Infant Mortality A Priority for Africa. 2018. https://intpolicydigest.org/2018/11/21/combatting-infant-mortality-a-priority-for-africa/.

[8] UNICEF, WHO, World Bank UDPD. UN Inter-agency Group for Child Mortality Estimation. The World Bank. 2017.

[9] United Nation. TRANSFORMING OUR WORLD: THE 2030 AGENDA FOR SUSTAINABLE DEVELOPMENT 2016.

[10] World Health Organization. Global Health Observatory (GHO) data. 2017. https://www.who.int/gho/child_health/mortality/neonatal_infant_text/en/.

[11] Mejía-Guevara I, Zuo W, Bendavid E, Li N, Tuljapurkar S (2019). Age distribution, trends, and forecasts ofunder-5 mortality in 31 sub-saharan africancountries: a modeling study. PLoS Med.

[12] Elkasabi M (2019). Calculating fertility and childhood mortality rates from survey data using the DHS.Rates R package. PLoS One.

[13] Janssen LDP (2008). The frailty model.

[14] Balan TA, Putter H (2020). A tutorial on frailty models. Stat Methods Med Res.

[15] Rathe M, Müller K, Sangild PT, Husby S (2014). Clinical applications of bovine colostrum therapy: a systematic review. Nutr Rev.

[16] Agha S (2000). The determinants of infant mortality in Pakistan. Soc Sci Med.

[17] Muluye S, Wencheko E (2012). Determinants of infant mortality in Ethiopia: a study based on the 2005 EDHS data. Ethiop J Heal Dev.

[18] Byberg S, Østergaard MD, Rodrigues A, Martins C, Benn CS, Aaby P (2017). Analysis of risk factors for infant mortality in the 1992-3 and 2002-3 birth cohorts in rural Guinea-Bissau. PLoS One.

[19] Vijay J, Patel KK (2020). Risk factors of infant mortality in Bangladesh. Clin Epidemiol Glob Heal.

[20] Rutstein SO (2005). Effects of preceding birth intervals on neonatal, infant and under-five years mortality and nutritional status in developing countries: evidence from the demographic and health surveys. Int J Gynecol Obstet.

[21] Pongou R (2013). Why is infant mortality higher in boys than in girls? A new hypothesis based on preconception environment and evidence from a large sample of twins. Demography..

[22] Alur *P. Sex* differences in nutrition, growth, and metabolism in preterm infants. Front Pediatrics. 2019;7 FEB:1–9. 10.3389/fped.2019.00022 PMC6374621 30792973

[23] Zhao D, Zou L, Lei X, Zhang Y (2017). Gender differences in infant mortality and neonatal morbidity in mixed-gender twins. Sci Rep.

[24] Uthman OA, Uthman MB, Yahaya I (2008). A population-based study of effect of multiple birth on infant mortality in Nigeria. BMC Pregnancy Childbirth.

[25] Ahrens KA, Thoma ME, Rossen LM, Warner M, Simon AE (2017). Plurality of birth and infant mortality due to external causes in the United States, 2000-2010. Am J Epidemiol.

[26] Lutz KF, Burnson C, Hane A, Samuelson A, Maleck S, Poehlmann J (2012). Parenting stress, social support, and mother-child interactions in families of multiple and singleton preterm toddlers. Fam Relat.

[27] Wenze SJ, Battle CL, Tezanos KM (2015). Raising multiples: mental health of mothers and fathers in early parenthood. Arch Womens Ment Health.

[28] Lawson DW, Mace R (2009). Trade-offs in modern parenting: a longitudinal study of sibling competition for parental care. Evol Hum Behav.

[29] Ezeh OK, Agho KE, Dibley MJ, Hall JJ, Page AN. Risk factors for postneonatal , infant , child and under-5 mortality in Nigeria : a pooled cross-sectional analysis. BMJ Open 2015;5:1–9. 10.1136/bmjopen-2014-006779 PMC4386230 25818271

[30] Miller SS, Lee HC, Gould JB (2011). Hypothermia in very low birth weight infants: distribution, risk factors and outcomes. J Perinatol.

[31] Belachew A, Tewabe T (2020). Neonatal sepsis and its association with birth weight and gestational age among admitted neonates in Ethiopia: systematic review and meta-analysis. BMC Pediatr.

[32] Edmond K, Newton S, Hurt L, Shannon CS, Kirkwood BR, Mazumder S (2016). Timing of initiation, patterns of breastfeeding, and infant survival: prospective analysis of pooled data from three randomised trials. Lancet Glob Heal.

[33] Berkat S, Sutan R (2014). The effect of early initiation of breastfeeding on neonatal mortality among low birth weight in Aceh Province, Indonesia: An Unmatched Case Control Study. Adv Epidemiol.

[34] Phukan D, Ranjan M, Dwivedi LK (2018). Impact of timing of breastfeeding initiation on neonatal mortality in India. Int Breastfeed J.

[35] Smith ER, Hurt L, Chowdhury R, Sinha B, Fawzi W, Edmond KM (2017). Delayed breastfeeding initiation and infant survival: a systematic review and meta-analysis. PLoS One.

[36] Uruakpa FO, Ismond MAH, Akobundu ENT (2002). Colostrum and its benefits: a review. Nutr Res.

[37] Friede A, Baldwin W, Rhodes PH, Buehler JW, Strauss LT, Smith JC (1987). Young maternal age and infant mortality: the role of low birth weight. Public Health Rep.

[38] Phaloprakarn YVTT (2017). Chadakarn, department. Inappropriate gestational weight gain among teenage pregnancies: prevalence and pregnancy outcomes. Int J Women's Health.

[39] Jama NA, Wilford A, Haskins L, Coutsoudis A, Spies L, Horwood C (2018). Autonomy and infant feeding decision-making among teenage mothers in a rural and urban setting in KwaZulu-Natal, South Africa. BMC Pregnancy Childbirth.

[40] Alam N (2000). Teenage motherhood and infant mortality in Bangladesh: maternal age-dependent effect of parity one. J Biosoc Sci.

[41] Rutstein Shea O. Further Evidence of the Effects of Preceding Birth Intervals on Neonatal,Infant,and Under-Five- Years Mortality and Nutritional Status in Developing Countries. Demogr Heal Res 2008;41:1–86. 10.1016/j.ijgo.2004.11.012 15820369

[42] Dadi AF (2015). A systematic review and meta-analysis of the effect of short birth interval on infant mortality in Ethiopia. PLoS One.

[43] Da Vanzo J, Hale L, Razzaque A, Rahman M (2008). The effects of pregnancy spacing on infant and child mortality in Matlab, Bangladesh: how they vary by the type of pregnancy outcome that began the interval. Popul Stud (NY).

[44] Sa A (2016). E U. maternal health care services access index and infant survival in Nigeria. Ethiop J Health Sci.

[45] Stanley WA, Brunner Huber LR, Laditka SB, Racine EF (2016). Association of type of birth attendant and place of delivery on infant mortality in sub-Saharan Africa. Afr Health Sci.

[46] Kiross GT, Chojenta C, Barker D, Tiruye TY, Loxton D (2019). The effect of maternal education on infant mortality in Ethiopia: a systematic review and meta-analysis. PLoS One.

[47] Fatima-Tuz-Zahura M, Mohammad KA, Bari W (2017). Log-logistic proportional odds model for analyzing infant mortality in Bangladesh. Asia-Pacific J Public Heal.

[48] Omariba DWR, Beaujot R, Rajulton F (2007). Determinants of infant and child mortality in Kenya: an analysis controlling for frailty effects. Popul Res Policy Rev.

[49] Monal R. Shroff, Paula L. Griffiths, Chirayath Suchindran, Balakrishna Nagalla, Shahnaz Vazir MEB. Does maternal autonomy influence feeding practices and infant growth in rural India? Monal. NIH Public Access 2011;73:1–7. 10.1016/j.socscimed.2011.05.040 PMC3164576 21742425

[50] Sartorius BKD, Sartorius K (2014). Global infant mortality trends and attributable determinants - an ecological study using data from 192 countries for the period 1990-2011. Popul Health Metrics.

[51] Ester PV, Torres A, Freire JM, Hernández V, Gil Á (2011). Factors associated to infant mortality in sub-Saharan Africa. J Public Health Africa.

[52] Kusneniwar GN, Mishra AK, Balasubramanian K, Reddy PS (2013). Determinants of infant mortality in a developing region in rural Andhra Pradesh. Natl J Integr Res Med.

[53] Ezeh OK, Agho KE, Dibley MJ, Hall J, Page AN (2014). The impact of water and sanitation on childhood mortality in Nigeria: evidence from demographic and health surveys, 2003–2013. Int J Environ Res Public Health.

[54] van Poppel F, van der Heijden C (1997). The effects of water supply on infant and childhood mortality: a review of historical evidence. Health Transit Rev.

