# Supplementary material for: Time to death and its associated factors among infants in sub-Saharan Africa using the recent demographic and health surveys: shared frailty survival analysis
Source: BMC Pediatr. 2021 Oct 4;21:433. doi: 10.1186/s12887-021-02895-7 (PMC8489062; doi:10.1186/s12887-021-02895-7)
Supplement: Supplementary file 1 — Additional file 1: Table A. Sample size for infant survival in SSA for each country. Table B. Infant mortality rate with their standard error for each SSA country. Figure A. Forest plot of the pooled estimate of IMR in urban inhabitants across SSA countries using the recent DHSs between 2010 to 2018, 2020. Figure B. Forest plot of the pooled estimate of IMR in rural inhabitants across SSA countries using the recent DHS between 2010 to 2018, 2020. Figure C. Forest plot of the pooled estimate of IMR by country income across SSA countries using the recent DHS between 2010 to 2018, 2020. [file 12887_2021_2895_MOESM1_ESM.docx]

Table S1: Sample size for infant survival in SSA for each Country.

| **SSA Countries with Recent DHS report from 2010/11 to 2017/18** | | | | |
| --- | --- | --- | --- | --- |
| **Regions** | **Country** | **DHS year** | **Sample size** | |
|  |  |  | **unweighted** | **Weighted** |
| East Africa countries | Burundi | 2016/17 | 3082 | 3178 |
|  | Comoros | 2012 | 779 | 803 |
|  | Ethiopia | 2016 | 2623 | 2735 |
|  | Kenya | 2014 | 4736 | 4418 |
|  | Malawi | 2015/16 | 3870 | 3892 |
|  | Mozambique | 2011 | 3007 | 3247 |
|  | Rwanda | 2014/15 | 1864 | 1894 |
|  | Tanzania | 2015/16 | 2384 | 2327 |
|  | Uganda | 2016 | 3949 | 3883 |
|  | Zambia | 2018 | 2297 | 2271 |
|  | Zimbabwe | 2015 | 1474 | 1526 |
| Central Africa countries | Angola | 2015/16 | 3517 | 3282 |
|  | Cameroon | 2011 | 3091 | 3125 |
|  | Chad | 2014/15 | 4710 | 4864 |
|  | The Democratic Republic of the Congo | 2013/14 | 4814 | 4757 |
|  | Republic of the Congo | 2011/12 | 2302 | 2039 |
|  | Gabon | 2012 | 1567 | 1354 |
| West Africa countries | Benin | 2017/18 | 3524 | 3551 |
|  | Burkina Faso | 2010 | 3980 | 4101 |
|  | Ivory Coast | 2011/12 | 2158 | 2038 |
|  | Gambia | 2013 | 2088 | 2010 |
|  | Ghana | 2014 | 1487 | 1425 |
|  | Guinea | 2018 | 2879 | 2835 |
|  | Liberia | 2013 | 2022 | 1689 |
|  | Mali | 2018 | 2495 | 2649 |
|  | Niger | 2012 | 3157 | 3462 |
|  | Nigeria | 2018 | 8697 | 8735 |
|  | Senegal | 2010/11 | 3105 | 2807 |
|  | Sierra Leone | 2013 | 3468 | 3612 |
|  | Togo | 2013/14 | 1667 | 1578 |
| Southern Africa countries | Lesotho | 2014 | 907 | 877 |
|  | Namibia | 2013 | 1269 | 1204 |
|  | South Africa | 2016 | 796 | 803 |
| **Total sample size** | | | **93,765** | **92, 977** |

Table S2: Infant mortality rate with their standard error for each SSA country.

| **Country** | **DHS year** | **IMR per 1000 live births (95%CI)** | **The standard error** |
| --- | --- | --- | --- |
| [Burundi](https://en.wikipedia.org/wiki/Burundi) | 2016/17 | 47.07 (41.82 – 52.32) | 2.68 |
| [Comoros](https://en.wikipedia.org/wiki/Comoros) | 2012 | 35.83 (27.09 – 44.57) | 4.46 |
| [Ethiopia](https://en.wikipedia.org/wiki/Ethiopia) | 2016 | 48.09 (41.45 – 54.73) | 3.39 |
| [Kenya](https://en.wikipedia.org/wiki/Kenya) | 2014 | 38.71 (34.83 – 42.59) | 1.98 |
| [Malawi](https://en.wikipedia.org/wiki/Malawi) | 2015/16 | 41.71 (37.65 – 45.77) | 2.07 |
| [Mozambique](https://en.wikipedia.org/wiki/Mozambique) | 2011 | 64.12 (57.81 – 70.43) | 3.22 |
| [Rwanda](https://en.wikipedia.org/wiki/Rwanda) | 2014/15 | 32.29 (27.88 – 36.70) | 2.25 |
| [Tanzania](https://en.wikipedia.org/wiki/Tanzania) | 2015/16 | 43.23 (38.13 – 48.33) | 2.60 |
| [Uganda](https://en.wikipedia.org/wiki/Uganda) | 2016 | 42.83 (38.91 – 46.75) | 2.00 |
| [Zambia](https://en.wikipedia.org/wiki/Zambia) | 2018 | 41.90 (36.88 – 46.92) | 2.56 |
| [Zimbabwe](https://en.wikipedia.org/wiki/Zimbabwe) | 2015 | 50.14 (43.97 – 56.31) | 3.15 |
| Angola | 2015/16 | 44.26 (38.91 – 49.61) | 2.73 |
| Cameroon | 2011 | 62.48 (56.60 – 68.36) | 3.00 |
| Chad | 2014/15 | 72.27 (66.31 – 78.23) | 3.04 |
| Democratic Republic of Congo | 2013/14 | 58.34 (53.40 – 63.28) | 2.52 |
| Republic of the Congo | 2011/12 | 39.38 (33.07 – 45.69) | 3.22 |
| Gabon | 2012 | 42.53 (35.08 – 49.98) | 3.80 |
| [Benin](https://en.wikipedia.org/wiki/Benin) | 2017/18 | 54.96 (49.98 – 59.94) | 2.54 |
| [Burkina Faso](https://en.wikipedia.org/wiki/Burkina_Faso) | 2010 | 65.13 (59.96 – 70.30) | 2.64 |
| [Ivory Coast](https://en.wikipedia.org/wiki/Ivory_Coast) | 2011/12 | 67.95 (58.74 – 77.16) | 4.70 |
| [Gambia](https://en.wikipedia.org/wiki/The_Gambia) | 2013 | 34.32 (28.58 – 40.06) | 2.93 |
| [Ghana](https://en.wikipedia.org/wiki/Ghana) | 2014 | 41.24 (34.52 – 47.96) | 3.43 |
| [Guinea](https://en.wikipedia.org/wiki/Guinea) | 2018 | 66.54 (59.17 – 73.91) | 3.76 |
| [Liberia](https://en.wikipedia.org/wiki/Liberia) | 2013 | 53.77 (46.48 – 61.06) | 3.72 |
| [Mali](https://en.wikipedia.org/wiki/Mali) | 2018 | 54.42 (48.17 – 60.67) | 3.19 |
| [Niger](https://en.wikipedia.org/wiki/Niger) | 2012 | 50.65 (45.06 – 56.24) | 2.85 |
| [Nigeria](https://en.wikipedia.org/wiki/Nigeria) | 2018 | 67.29 (62.80 – 71.78) | 2.29 |
| [Senegal](https://en.wikipedia.org/wiki/Senegal) | 2010/11 | 46.74 (42.00 – 51.48) | 2.42 |
| [Sierra Leone](https://en.wikipedia.org/wiki/Sierra_Leone) | 2013 | 92.38 (85.30 – 99.46) | 3.61 |
| [Togo](https://en.wikipedia.org/wiki/Togo) | 2013/14 | 48.52 (42.33 – 54.71) | 3.16 |
| [Lesotho](https://en.wikipedia.org/wiki/Lesotho) | 2014 | 59.49 (49.55 – 69.43) | 5.07 |
| [Namibia](https://en.wikipedia.org/wiki/Namibia) | 2013 | 38.88 (32.12 – 45.64) | 3.45 |
| [South Africa](https://en.wikipedia.org/wiki/South_Africa) | 2016 | 35.47 (26.63 – 44.51) | 4.51 |

Figure S1: Forest plot of the pooled estimate of IMR in urban inhabitants across SSA countries using the recent DHSs between 2010 to 2018, 2020.

Figure S2: Forest plot of the pooled estimate of IMR in rural inhabitants across SSA countries using the recent DHS between 2010 to 2018, 2020.

Figure S3: Forest plot the pooled estimate of IMR by country income across SSA countries using the recent DHS between 2010 to 2018, 2020.
